# Supplementary material for: Reducing Seed Shattering in Weedy Rice by Editing SH4 and qSH1 Genes: Implications in Environmental Biosafety and Weed Control through Transgene Mitigation
Source: Biology (Basel). 2022 Dec 14;11(12):1823. doi: 10.3390/biology11121823 (PMC9776087; doi:10.3390/biology11121823)
Supplement: Supplementary file 1 [file biology-11-01823-s001.zip › Supplementary Tables S1 and S2 .pdf]

Table S1. Primers for the amplification and verification of the *SH4*- and *qSH1*-gene editing target sequencing

Gene	Primer	Sequence (5'–3')
SH4	SH4-V-F	ACCAAACGCCTCAGCTTGCCTT
	SH4-V-R	TAGTCGCGGACCTTCTTGTAGT
qSH1	qSH1-V -F	TATCTCCACTCTCTCCAGGGCC
	qSH1-V -R	ACGAGGACGCGTACGAGTAGAAC

Table S2. Primers for the real-time PCR examination on the seed shattering-related genes in the gene-edited and parental weedy rice lines.

Gene	Primer	Sequence (5'–3')
CPL1	CPL1-F	GCCAAGAAGAAGAAGTCTGT
	CPL1-R	GACCACCTGAGAAACTTCATT
SH4	SH4-F	GAACTACTGCTGGAAGAACG
	SH4-R	TCTTGTAGTCGCGGAGGA
SHAT1	SHAT1-F	GATTGTGGCAAGCAGGTC
	SHAT1-R	CTCGTAATCCTCCAAGCTGAA
OsSH1	OsSH1-F	ATGCTAAACATCGTGACCG
	OsSH1-R	GGAGTGCTTGGACCAATC
OsSH15	OsSH15-F	TTGGTTCTTCTGAGGATGACA
	OsSH15-R	GCTGAAACTTGAGCTCCTTAT
qSH1	qSH1-F	GTCATGGTCATGGTCATGC
	qSH1-R	AGGACGCGTACGAGTAGAA
SH5	SH5-F	CCAGCAACTCCAAGCTGTA
	SH5-R	TTGTCCTAAGAGCCATGGAAG
SH8	SH8-F	TCTACGAGTTCTGCAGCAC
	SH8-R	TGTTTCGGGTCCTGCGTAA
ZEP	ZEP-F	GGTGCTGATGGAATATGGTC
	ZEP-R	AAAGTCTGCAATGCCAGT
NCED1.1	NCED1.1-F	GAAAGCCGGGTTGATCAC
	NCED1.1-R	TCGAGAAGGTGCGCATCAA
NCED1.2	NCED1.2-F	ATGCAAAGGATTTGCCCTG
	NCED1.2-R	AGGAGGGATGTAGGAAAGC
SDR1	SDR1-F	CTCGACATCCTGGTGAACA
	SDR1-R	CGTAGAAGTTGGTCCTGAGCA
SDR	SDR -F	TAAGCCAGCTGATGCACC
	SDR -R	ATGGGAAGAGATGGGAACC
PYL3	PYL3 -F	GTCCACCTGGTTTGGTCT
	PYL3 -R	TTTCATCTCACACCGGCTC
PYL4	PYL4 -F	CGTCGTGGTGGAGTCCTA
	PYL4 -R	GGTATTGGCGAGAGACTGG
PP2C39	PP2C39 -F	TCCAATGATCACAGCATCAGT
	PP2C39 -R	TATCCAAAGCTGACGTTGAGA
SAPK5	SAPK5 -F	ATCGAGAGAGGGAACAGG
	SAPK5 -R	ATTATGTTCGGGTGACGCA
NZIP23	NZIP23 -F	CAGACGCCGATGTTGTTC
	NZIP23 -R	GAGACCAGCCCATTTCCC
PME34	PME34 -F	GGAACACCTCAGCAACAG
	PME34 -R	TGGAGTTGGTTGTGGACG
PG	PG -F	TTCGTCTAAGAGCCATCTCAT
	PG -R	TACCCTGTGGTAACTTCGG
XTN28	XTN28 -F	TGGAGAGTGCAGACCAAC
	XTN28 -R	GAAATCCTCCGTCGGATCA
MAN4	MAN4 -F	GGGCTCAACCTGGCAAGAA
	MAN4 -R	GGAACATGGTCTCGTTGTAG
Ubi	*Ubi* -F	GATCAAGAACTAGAGCGTCAC
	Ubi -R	GGGAGATAACAACGGAAGC
