# Supplementary figures and images for: Reducing Seed Shattering in Weedy Rice by Editing SH4 and qSH1 Genes: Implications in Environmental Biosafety and Weed Control through Transgene Mitigation
Source: Biology (Basel). 2022 Dec 14;11(12):1823. doi: 10.3390/biology11121823 (PMC9776087; doi:10.3390/biology11121823)

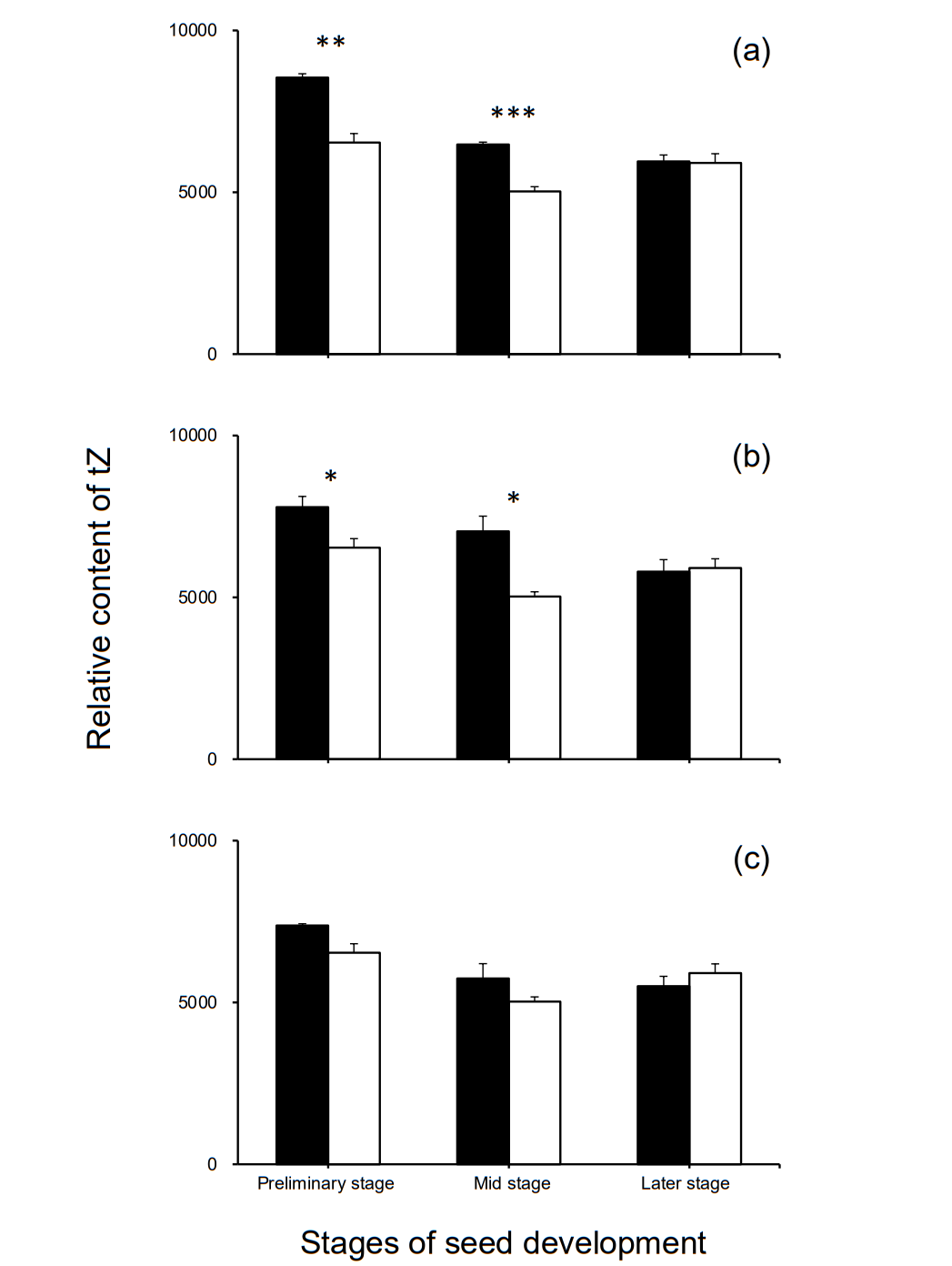

Supplement: Supplementary file 1 [file biology-11-01823-s001.zip › Figure S1.tif]

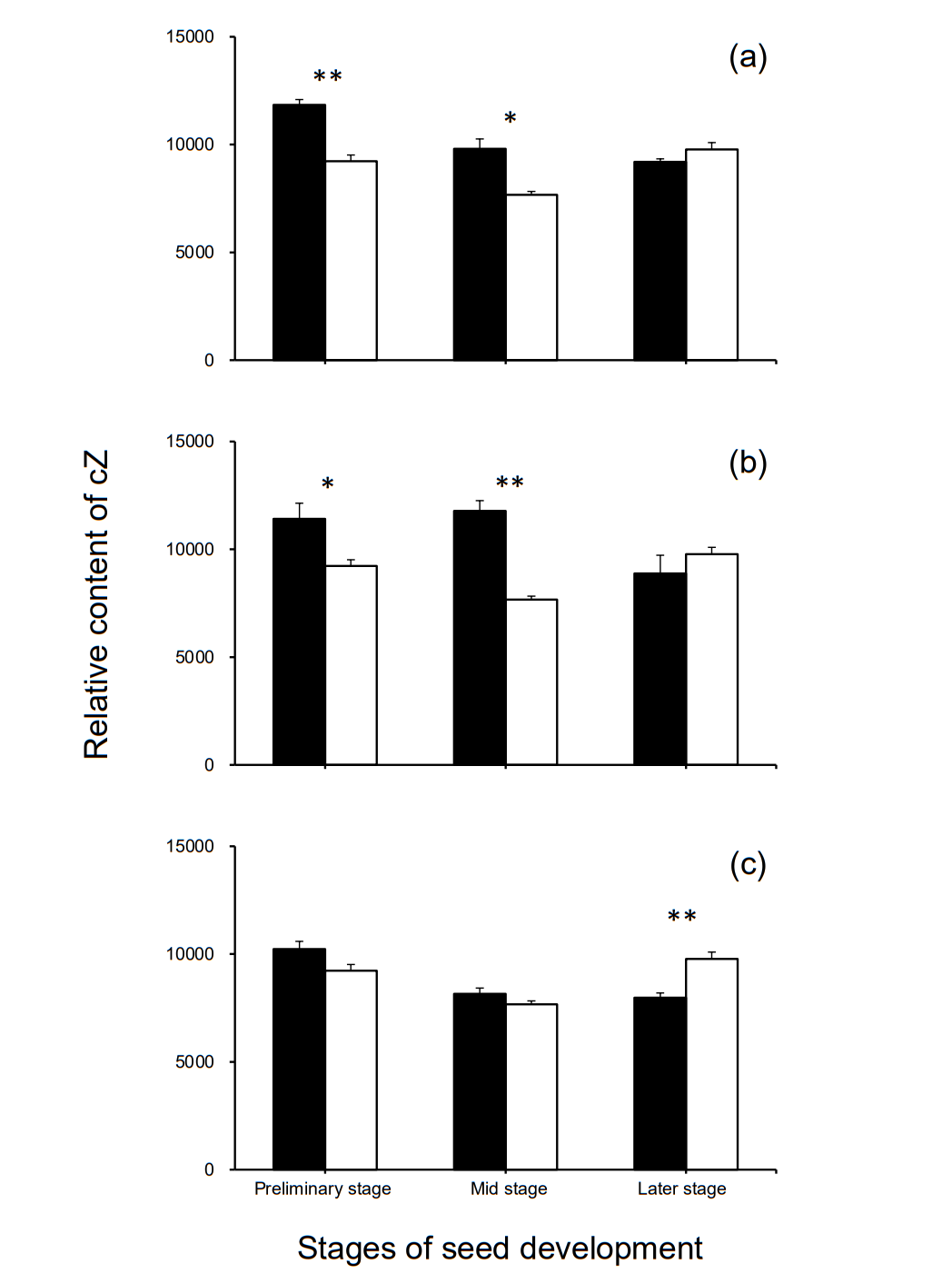

Supplement: Supplementary file 1 [file biology-11-01823-s001.zip › Figure S2.tif]

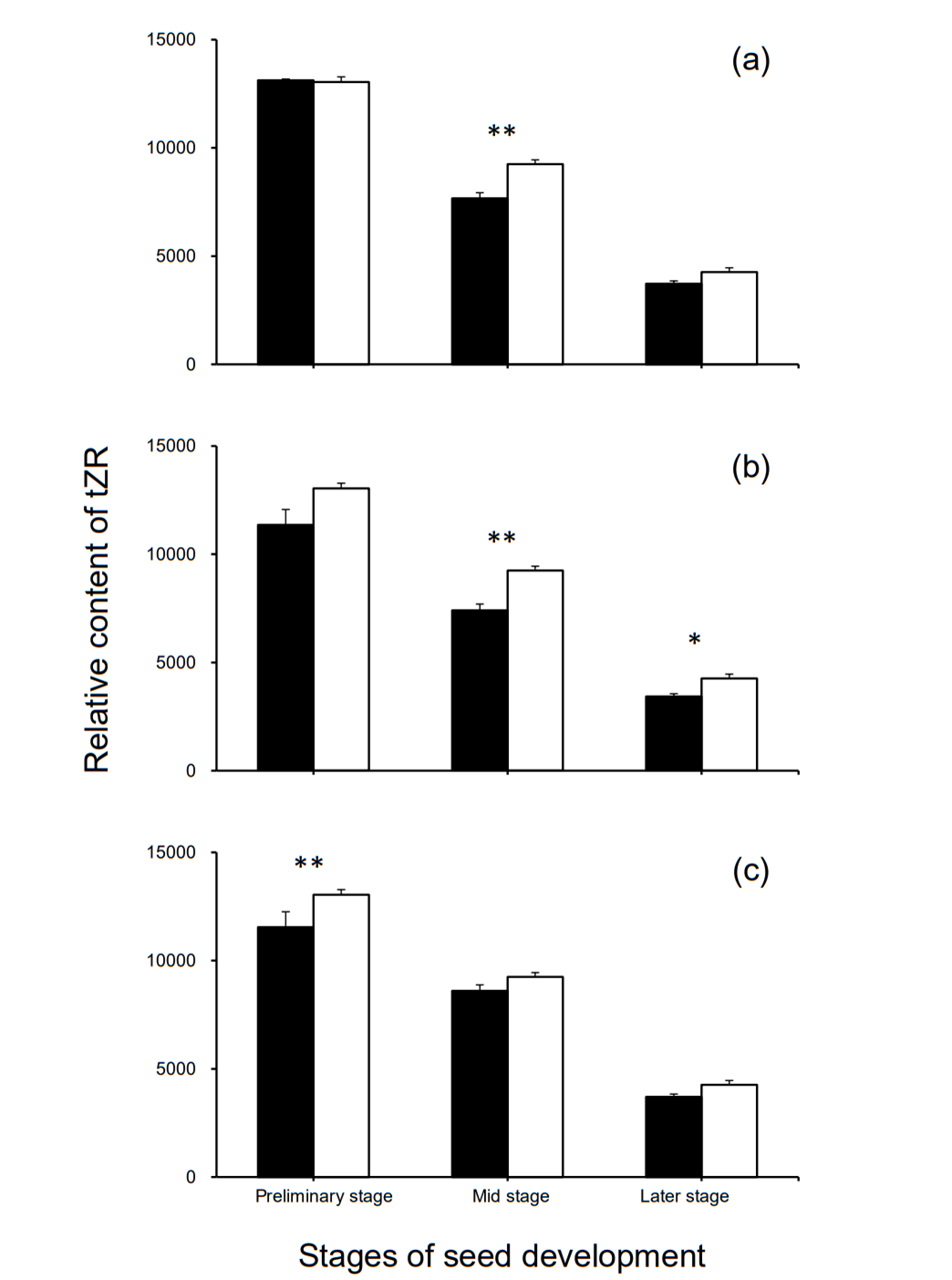

Supplement: Supplementary file 1 [file biology-11-01823-s001.zip › Figure S3.tif]

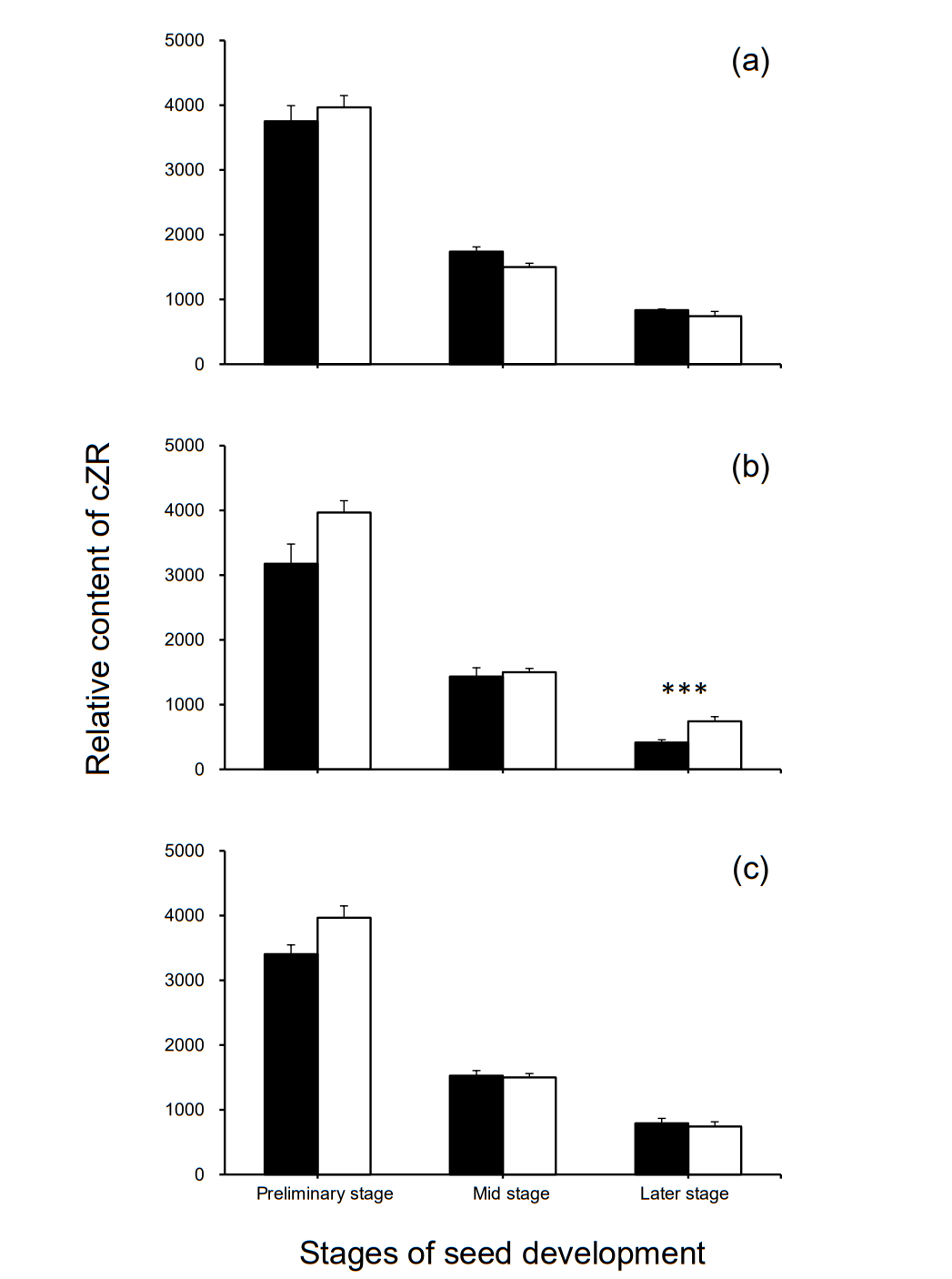

Supplement: Supplementary file 1 [file biology-11-01823-s001.zip › Figure S4.tif]

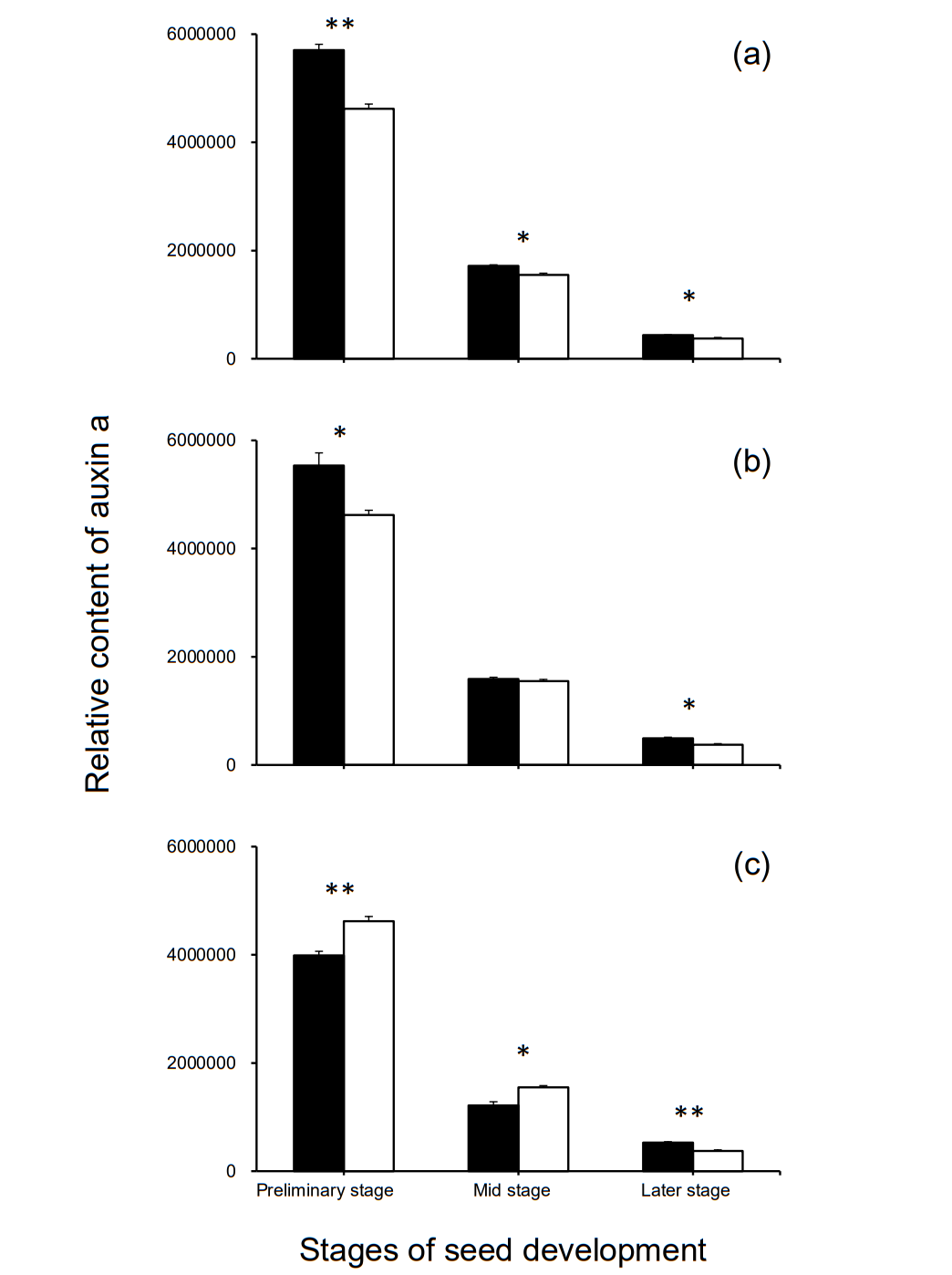

Supplement: Supplementary file 1 [file biology-11-01823-s001.zip › Figure S5.tif]

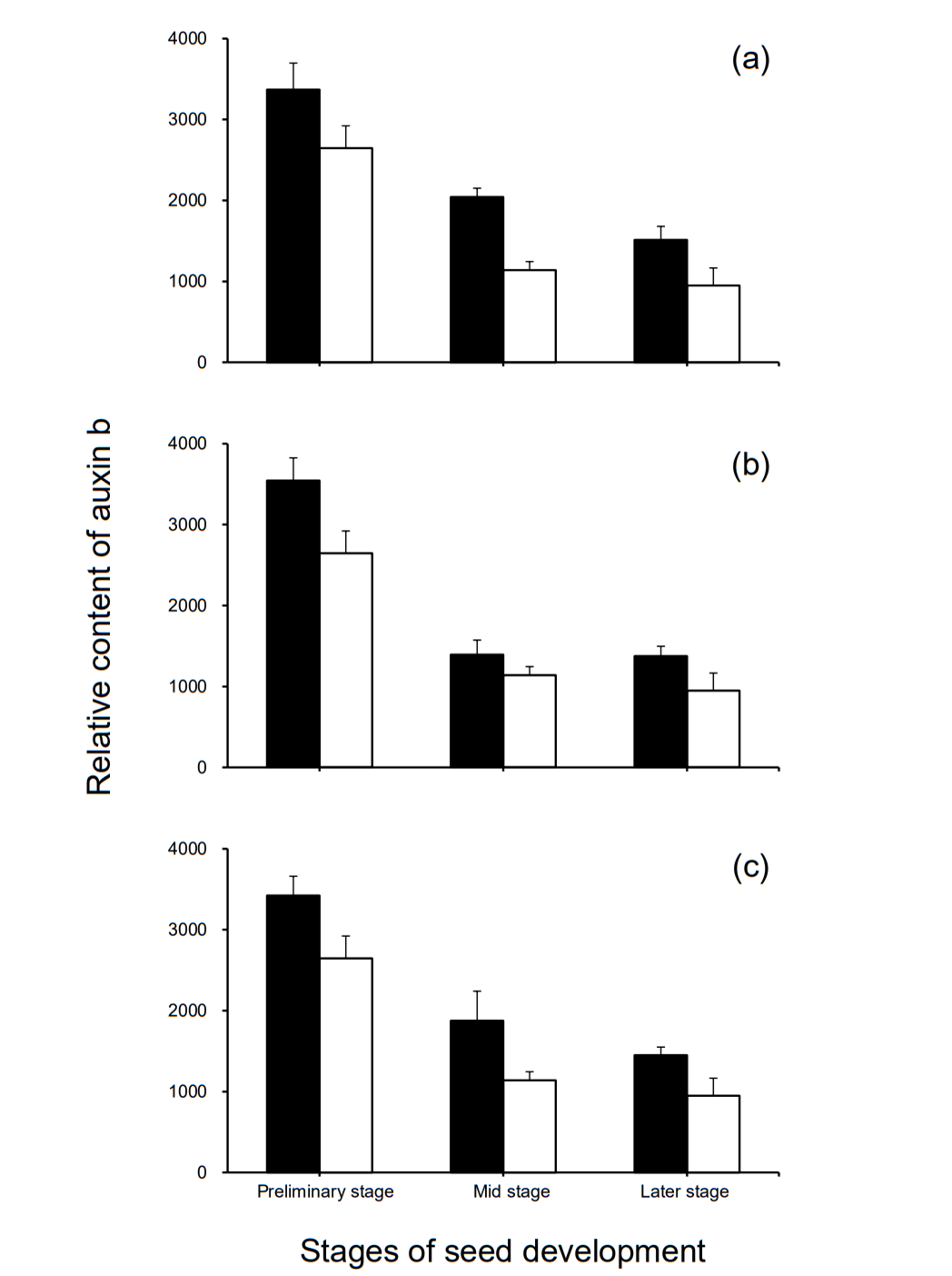

Supplement: Supplementary file 1 [file biology-11-01823-s001.zip › Figure S6.tif]
